# Exploring the impact of AI-assisted practice applications on music learners’ performance, self-efficacy, and self-regulated learning

**DOI:** 10.3389/fpsyg.2025.1675762

**Published:** 2025-10-13

**Authors:** Jiayi Ou, João Nogueira, Chao Qin

**Affiliations:** ^1^CESEM/NOVA FCSH, Faculty of Social Sciences and Humanities, NOVA University of Lisbon, Lisbon, Portugal; ^2^School of Education, Yunnan Minzu University, Kunming, China

**Keywords:** AI-assisted app, musicians’ self-efficacy, musicians’ performance, self-regulated learning, quasi-experimental study

## Abstract

**Introduction:**

Despite significant advancements in artificial intelligence (AI) applications across various disciplines, research on AI’s psychological impacts in music learning contexts remains limited. This study explores the effects of AI-assisted practice apps on violin students’ self-efficacy, performance outcomes and Self-Regulated Learning (SRL).

**Methods:**

A four-month quasi-experimental study was conducted with 40 violin majors from a conservatory in South China. All participants received identical classroom instruction and maintained equivalent daily practice time, but the experimental group (*n* = 20) used AI-assisted practice app while the control group (*n* = 20) practiced using regular practice methods.

**Results:**

Mixed-effects modelling revealed differentiated impacts on self-efficacy dimensions: while the control group experienced natural decline in Music Learning Self-Efficacy (MLSE) as task difficulty increased, AI intervention enabled the experimental group to maintain stable learning confidence. More notably, the experimental group achieved significant improvements in Music Performance Self-Efficacy (MPSE) with large effect sizes, indicating that AI-assisted practice app possesses distinct advantages in enhancing performance confidence. In terms of performance outcomes, the experimental group demonstrated significant improvement while the control group showed a declining trend. Thematic analysis revealed that AI-assisted practice apps support self-regulated learning (SRL) across three critical phases: providing goal-setting and strategic planning support during the forethought phase, facilitates self-monitoring and self-control during the performance phase, and enabling objective evaluation and strategic adjustment during the self-reflection phase.

**Discussion:**

This study enriches understanding of self-efficacy theory in AI technology-enhanced learning environments and demonstrates AI technology’s educational value in instrumental music learning.

## 1 Introduction

In recent years, the rapid advancement of artificial intelligence (AI) technology in education has brought revolutionary changes to traditional teaching models (Chen et al., 2020). This transformation is particularly prominent in music education, with the Apple App Store offering nearly 50 applications that utilize AI for personalized feedback and adaptive music learning, among which “Violin by Trala” has attracted over 400,000 users across 193 countries.

In the field of music education, instrumental learning represents a complex skill acquisition process requiring sustained practice and immediate feedback, making it an important application scenario for AI technology. Through advanced tools such as audio processing and pattern recognition, AI can provide real-time feedback on pitch accuracy, rhythm, and overall performance quality (Evin, 2024; Lu, 2025). These AI-assisted systems have the potential to reshape music learners’ practice patterns.

However, the complexity of instrumental learning extends beyond technical considerations. Given music’s inherently performative nature, learners must demonstrate their acquired skills in both learning and performance contexts, which makes instrumental learning outcomes closely related to learners’ psychological and cognitive factors (Oliveira et al., 2021). Within this complex learning process, psychological and cognitive factors such as self-regulated learning (SRL) abilities and self-efficacy have been demonstrated as significant predictors of musical performance (McPherson et al., 2017; Zarza-Alzugaray et al., 2020; Dong and Gedvilienė, 2025). Consequently, the potential impact of AI technology on learners’ cognitive processes, particularly its influence on and cultivation of self-efficacy and self-regulatory abilities, emerges as a critical area of inquiry.

Despite the gradual implementation of AI-assisted systems in instrumental teaching practice, existing research exhibits notable limitations. On one hand, most studies focus on technical feature development, lacking rigorous validation of AI tools’ effects on self-efficacy and performance outcomes. On the other hand, the mechanisms through which AI enhances performance by supporting learners’ self-regulatory processes remain underexplored.

Therefore, this study employs a mixed-methods quasi-experimental design to address these research gaps. While quantitatively examining the effects of AI-assisted practice app on self-efficacy and performance, the study also emphasizes the underlying mechanisms of its impact on learners’ self-regulatory processes through qualitative analysis. The findings will provide empirical evidence for the practical application of AI in music education and deepen the understanding of technology-enhanced learning environments from a self-regulated learning perspective.

## 2 Literature review

### 2.1 Music self-efficacy and performance

Self-efficacy is defined as an individual’s self-assessment and judgment of their ability to perform a specific behavior (Bandura, 1977). It is a core factor in self-regulated learning (SRL), as it not only drives learners to actively regulate their learning processes but also significantly impacts the persistence and effectiveness of learning (Zimmerman, 2002). Self-efficacy affects the learning process in various ways, primarily by determining the level of effort and persistence a learner invests (Schunk and Mullen, 2012).

Applying these foundational principles to the domain of music learning, self-efficacy becomes a critical predictor of success in complex instrumental tasks. Learners with higher self-efficacy are more likely to set clear goals, employ diverse learning strategies, and improve their learning efficiency through continuous reflection and adjustment when faced with complex tasks (McPherson and McCormick, 2006). They are more willing to confront technical challenges, believing that they can overcome obstacles through practice (Gale et al., 2021; Chang et al., 2022). For instance, a study on piano learners found a significant positive correlation between self-efficacy and practice time, practice efficiency, as well as their final performance level (Cheng and Southcott, 2023; Kandemir and Yokuş, 2023). Furthermore, within the music practice context, self-efficacy enhances learners’ self-regulatory abilities through interactions with self-assessment and feedback mechanisms. By engaging in repeated practice, adjusting goals, and receiving feedback, learners accumulate positive success experiences, which in turn boost their self-efficacy (Miksza, 2015; Chen, 2024).

Most studies indicate a strong positive correlation between self-efficacy and musical performance success, where self-efficacy reliably predicts learners’ musical proficiency (Chen, 2024; Wang and Li, 2024; Zelenak, 2024). For young music students, self-efficacy is a crucial precursor to skill improvement and performance success (MacAfee, 2021). Self-efficacy is not only an individual’s judgment of their musical abilities but also a key factor in fostering confidence and motivation in music learning and performance (Jiang, 2024; Zelenak, 2024).

Research on self-efficacy highlights its situational nature, with efficacy beliefs varying across tasks (Ahola et al., 2023; Coluccio et al., 2024). This distinction is particularly critical in music, leading to two specific types of self-efficacy: music learning self-efficacy (MLSE) and music performance self-efficacy (MPSE) (Ritchie and Williamon, 2011). MLSE refers to a musician’s confidence in learning and preparing music, including overcoming challenges and practicing persistently, while MPSE focuses on confidence during performance, handling challenges, and managing pressure (Nenadic, 2023). Both types are essential for a musician’s motivation, performance, and professional development.

Researchers have developed scales to measure music self-efficacy in areas like jazz (Regier, 2022) and orchestral performance (MacRitchie and Garrido, 2019), enabling assessment in music. However, most studies treat self-efficacy as a singular concept, limiting its practical use for educators. Recognizing the distinction between learning an instrument and performing on stage (Nelson, 2023), Ritchie and Williamon (2011) developed the General Musical Self-Efficacy Scale, which differentiates between Music Learning Self-Efficacy (MLSE) and Music Performance Self-Efficacy (MPSE), offering a more comprehensive tool for assessment. Our study adopted this scale.

### 2.2 SRL in music education

Rooted in Bandura’s (1986) social cognitive theory, self-Regulated Learning (SRL) refers to the process by which learners actively manage their own learning behaviors through strategies such as goal setting, planning, monitoring, and reflection (Zeidner and Stoeger, 2019). Within the domain of music, a growing body of research has focused on how SRL helps students (Antonini Philippe et al., 2020; Daubney and Fautley, 2020; Wang and Li, 2024) and professional musicians (López-Íñiguez and McPherson, 2020; Gaunt et al., 2021) enhance their practice and improve performance outcomes (López-Íñiguez and McPherson, 2024).

The three-phase model of SRL, originally developed by Zimmerman’s (2002) as a general framework for learning, has been specifically adapted and applied to music education by researchers such as Hatfield et al. (2017). This model provides a powerful theoretical framework for understanding how music learners manage their practice activities. In the Forethought Phase, music learners analyze the task and set goals, such as addressing technical difficulties in a particular passage or enhancing musical expressiveness, while also creating a specific practice plan. The success of this phase relies on the learner’s motivation and clear understanding of the task (Chen, 2024). During the Performance Phase, they execute their plan while assessing progress through auditory feedback or external tools. They would continually adjust intonation, technique, rhythm, and expression to achieve their goals. The key to this phase lies in active self-monitoring and strategy adjustment (Spahn et al., 2023). For example, music learners might resolve complex technical issues by practicing in sections or playing slowly. During this process, external feedback (such as teacher guidance) can help learners identify problems more precisely. Antonini Philippe et al. (2020) found that music learners significantly reduced technical error rates by identifying pitch deviations through playback recordings and instantly switching practice strategies (such as adjusting fingering or rhythm). In the Self-Reflection Phase, musicians analyze the playback of recordings, evaluating the achievement of previous goals, or keeping a practice log to reflect on learning progress. López-Íñiguez and McPherson (2020) conducted longitudinal research that showed professional musicians optimize their long-term practice plans through metacognitive reflection by writing practice logs (recording emotional states and technical difficulties).

Research shows that music learners employ a variety of self-regulation strategies in their practice, such as setting clear practice goals, creating plans, monitoring progress, and reflecting on performance (Nguyen and Nguyen, 2024; Dos Santos Silva and Marinho, 2025). These strategies help music learners break down tasks more effectively when facing technical challenges and maintain efficiency and focus during independent practice. In particular, for high-level musicians, self-regulation ability is considered an important factor in determining the quality of practice and performance (Taylor, 2021).

Although the importance of SRL in music education is widely recognized, researchers generally point out that many music learners lack systematic guidance or feedback support during independent practice (Roberts, 2024). Traditional music education models typically rely on regular teacher guidance, but during the independent practice phase, learners often struggle to effectively implement SRL strategies, limiting the improvement of practice efficiency and learning outcomes (Coetzer, 2024).

### 2.3 AI in music education

With technological advancements, recent educational research has increasingly focused on the potential of digital tools and artificial intelligence to support SRL. For example, AI-driven learning platforms can assist learners in setting goals through personalized suggestions (Sajja et al., 2024), provide immediate feedback during the learning process (Hasnine et al., 2024), and support reflection through data analysis (Tsao and Nogues, 2024). However, research on the application of advanced, multi-faceted AI in music education is still limited. While some studies have explored the use of specific software for intonation feedback for 10–11 years old novice learners, revealing positive impacts on their self-regulation strategies (López-Calatayud and Tejada, 2024) and self-efficacy (López-Calatayud and Tejada, 2023), the exploration of how integrated AI apps can support the entire SRL cycle for advanced music majors remains a relatively unexplored area.

Current AI research in music education mainly focuses on areas such as music composition, virtual performances, music tutoring systems, and interactive learning (Lin and Yunus, 2024). Music composition platforms based on generative models, such as GPT, can automatically generate melodies or accompaniments, providing students with creative inspiration and a space for experimentation (Candusso, 2024; Banar, 2025). Music online teaching platforms, such as Music Major, can model the teaching process, analyze it, and conduct systematic research, enabling informed decisions for the accurate allocation of music education and resources, particularly in instrumental education such as piano and violin instruction (Jamshidi et al., 2021; Wang, 2022; Arum and Jacob, 2024). Despite some evidence suggesting that AI applications can improve the effectiveness of music education (Dai, 2021), there remains a notable gap in research examining how AI-assisted practice tools influence key psychological constructs such as self-efficacy and self-regulated learning—factors that are crucial for sustained musical development.

## 3 Research questions

This study addresses these gaps by investigating the effects of AI-assisted practice apps using a mixed-methods approach. Participants were recruited through convenience sampling, focusing specifically on violin students. This targeted approach ensured both practical feasibility and consistency in the educational context, since different musical instruments involve distinctly different teaching methods and learning processes.

Our research questions are as follows:

RQ1: What are the effects of the AI-assisted practice app on the music learning self-efficacy (MLSE) of violin students?

RQ2: What are the effects of the AI-assisted practice app on the music performance self-efficacy (MPSE) of violin students?

RQ3: What are the effects of the AI-assisted practice app on the performance outcomes of violin students?

RQ4: In what ways does the AI-assisted practice app influence the self-efficacy and performance of violin students within the self-regulated learning (SRL) framework?

This study will help explore the potential of AI-assisted practice apps in improving music students’ self-efficacy and self-regulated learning, while also offering valuable insights for future music teaching strategies.

## 4 Method

### 4.1 Participants

An *a priori* power analysis was conducted using G*Power 3.1.9.7 (Faul et al., 2007) to approximate the minimum required sample size for the planned mixed-effects model analysis. Using the closest available design in G*Power (a 2 × 2 mixed ANOVA with repeated measures ANOVA, within-between interaction), the analysis was based on a medium effect size (*f* = 0.25), an alpha level of 0.05, power (1 − β) of 0.80, two groups, two measurement points, a correlation among repeated measures of 0.50, and a nonsphericity correction *ε* = 1. The results indicated that a total sample size of 34 participants (17 per group) would be sufficient to detect the hypothesized effect.

This study was conducted at a public conservatory in South China. Forty violin performance majors were recruited through convenience sampling and divided into the experimental group (*n* = 20) and control group (*n* = 20). The gender distribution and grade levels of the control and experimental groups are shown in Tables 1, 2, respectively.

**Table 1:** Gender distribution of control and experimental groups (*N* = 40).

Group	Male	Female	Sum
CG	7	13	20
EG	7	13	20
Total	14	26	40

**Table 2:** Grade distribution of control and experimental groups (*N* = 40).

Group	Freshman	Sophomore	Junior	Senior	Master’s Year 1	Master’s Year 2	Master’s Year 3	SUM
CG	5	4	4	3	1	2	1	20
EG	7	4	3	2	1	2	1	20
Total	12	8	7	5	2	4	2	40

To ensure homogeneity, all participants, aged 19 to 25, had more than 10 years of violin learning experience, maintained consistent daily practice routines (3–5 h per day). Screening during recruitment confirmed that while a few participants had used traditional practice aids (e.g., a physical metronome), none had prior experience with any software that provides real-time analysis or interactive feedback on their performance. Informed consent was obtained from all participants before the study commenced.

To assess the baseline comparability of the two groups, we conducted independent samples t-tests on the academic performance and levels of self-efficacy between the control and experimental groups, as shown in Table 3. The data indicated no significant differences between groups, suggesting similar starting points before the experiment.

**Table 3:** Independent samples *t*-test of pre-test between and control experimental groups.

Variable	Group	*n*	M	SD	Levene’s test		*t*-test			
					F	sig	*t*	*df*	*p*	Cohen’s d
MLSE	CG	20	5.45	0.92	2.25	0.14	−0.71	38	0.482	0.18
	EG	20	5.20	1.28						
MPSE	CG	20	4.72	1.11	1.91	0.18	0.39	38	0.701	−0.12
	EG	20	4.88	1.50						
Performance	CG	20	84.7	1.56	0.84	0.37	0.96	38	0.342	−0.30
	EG	20	85.25	2.02						

The experimental group received free access to an AI-assisted violin practice application during their regular after-class practice sessions. The control group continued their habitual individual practice methods without AI technological support.

Crucially, the total amount of practice time outside of class remained consistent with each participant’s pre-study routine across both groups. No additional practice time was introduced, and no changes were made to the participants’ regular schedules. The only difference between the two groups was the integration of the AI-assisted practice tool in the experimental group.

### 4.2 AI-assisted practice app

We provided the students in experimental group with an AI-assisted practice app called Violy, which was installed on their own personal devices (smartphones or tablets). It supports both Apple iOS and Android systems. Violy offers practice sessions for various instruments including piano, violin, saxophone, flute, and trumpet. The app utilizes Score Following technology, which employs AI and machine learning algorithms to analyze players’ intonation, rhythm, and performance techniques, supported by the OLTW and GMM algorithm.

**Figure 1:**
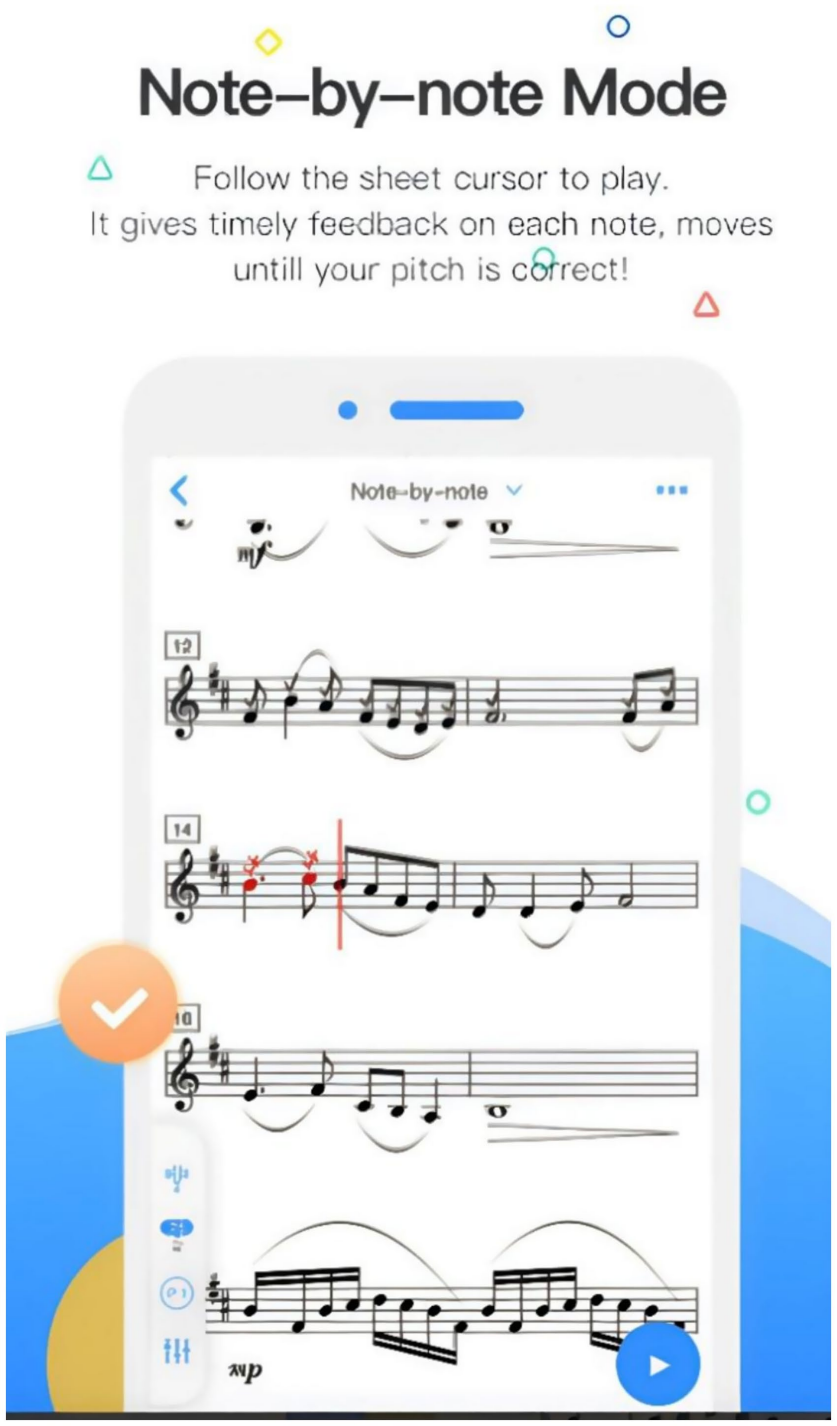
“Note-by-Note” interface in the Violy app. Screenshot courtesy of Violy - https://violy.app/blog/.

**Figure 2:**
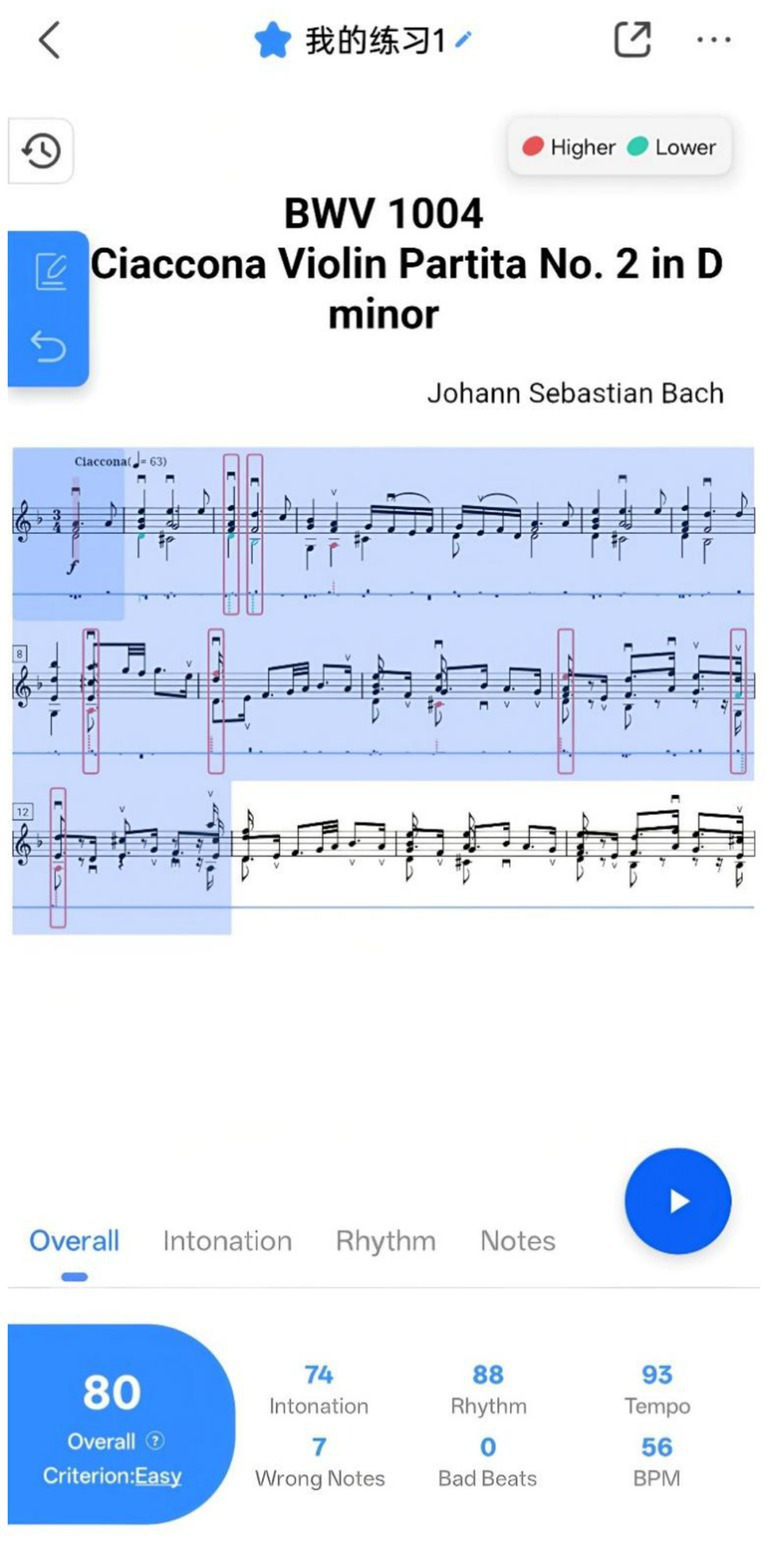
Audition performance scoring interface in the Violy app. Screenshot courtesy of Violy - https://violy.app/blog/.

Violy provides various features for music learners, including Daily Challenge, Music Cloud, Video Demonstrations, Accompaniment, Audition Report, and Note-by-Note (see Table 4 for details). The two core functionalities are Note-by-Note and Audition. Note-by-Note (Figure 1) helps learners quickly familiarize themselves with musical pieces by evaluating pitch accuracy in real-time, displaying visual comparisons on the digital score to help learners correct their pitch immediately. Audition (Figure 2) serves as Violy’s primary evaluation mode, providing comprehensive feedback on overall performance, intonation, rhythm, and notes after each practice session. Through the feedback interface, learners can review detailed analysis of each note’s intonation and rhythm while comparing their performance with reference recordings.

**Table 4:** Violy software feature list.

Functions	Introduction
Daily Challenge	Automatically recorded practice time, recording every second of the progress!
Music Cloud	Digital sheet music from original sheet music books/syllabus. Also, Users can upload own sheet music through Creator Studio.
Video Demonstrations	By observing the demonstrations of professional performers, practicing the instrument becomes more intuitive. While playing, the score will scroll along with the video
Accompaniment	Find various digital music scores, play along with accompaniments and ensembles and customize the practice! Tempo, volume, and parts can be user-defined.
Audition Report	Al understands the sound of musical instruments and scores performances, providing timely feedback to improve practice efficiency.
Note-by-Note	Compared to the “Audition” mode, “Note-by-note” is more like a mode for practicing. After receiving a sound in correct pitch, a checkmark would be ticked on the corresponding note. It helps users to get familiar with the music in a short time.

Function names may vary by software version.

### 4.3 Instruments

Music self-efficacy was assessed using the scale developed by Ritchie and Williamon (2011). It consisting of two subscales: the music learning self-efficacy subscale (MLSE; 11 items) and the music performance self-efficacy subscale (MPSE; 9 items). Representative items included: “I am confident that I can successfully learn the music for this performance” (MLSE) and “I am confident that I can succeed in the performance” (MPSE). Responses were recorded on a 7-point Likert scale, ranging from 1 (“Strongly Disagree”) to 7 (“Strongly Agree”). The original study reported Cronbach’s α coefficients of 0.82 for MLSEs and 0.78 for MPSEs, demonstrating good internal reliability (Ritchie and Williamon, 2011). The subscales were translated into Chinese by a master’s graduate in English translation, then reviewed by three experts in violin performance, music education, and educational psychology. Five violin students from the conservatory tested the translated items for semantic clarity and comprehension. To ensure translation accuracy, another master’s graduate in English conducted back-translation, and the results were compared with the original versions to verify consistency.

Additionally, we included a fill-in-the-blank item to collect participants’ music performance scores. In the pre-test, participants reported their violin performance scores from the previous semester’s final exam, and in the post-test, they reported their scores from the current semester’s final exam. This self-report procedure served to document participant consent for the use of their official grades; to ensure the accuracy of this performance data, each score was subsequently verified by the first author against the official records with permission from the examining instructors, who were blind to the participants’ group assignments.

The experimental and control groups showed similar distributions across academic years (see Table 2). All participants completed the same standardized year-level examinations, which were graded by an expert committee from the Orchestral Department using consistent and uniform assessment criteria.

### 4.4 Research design and procedure

**Figure 3:**
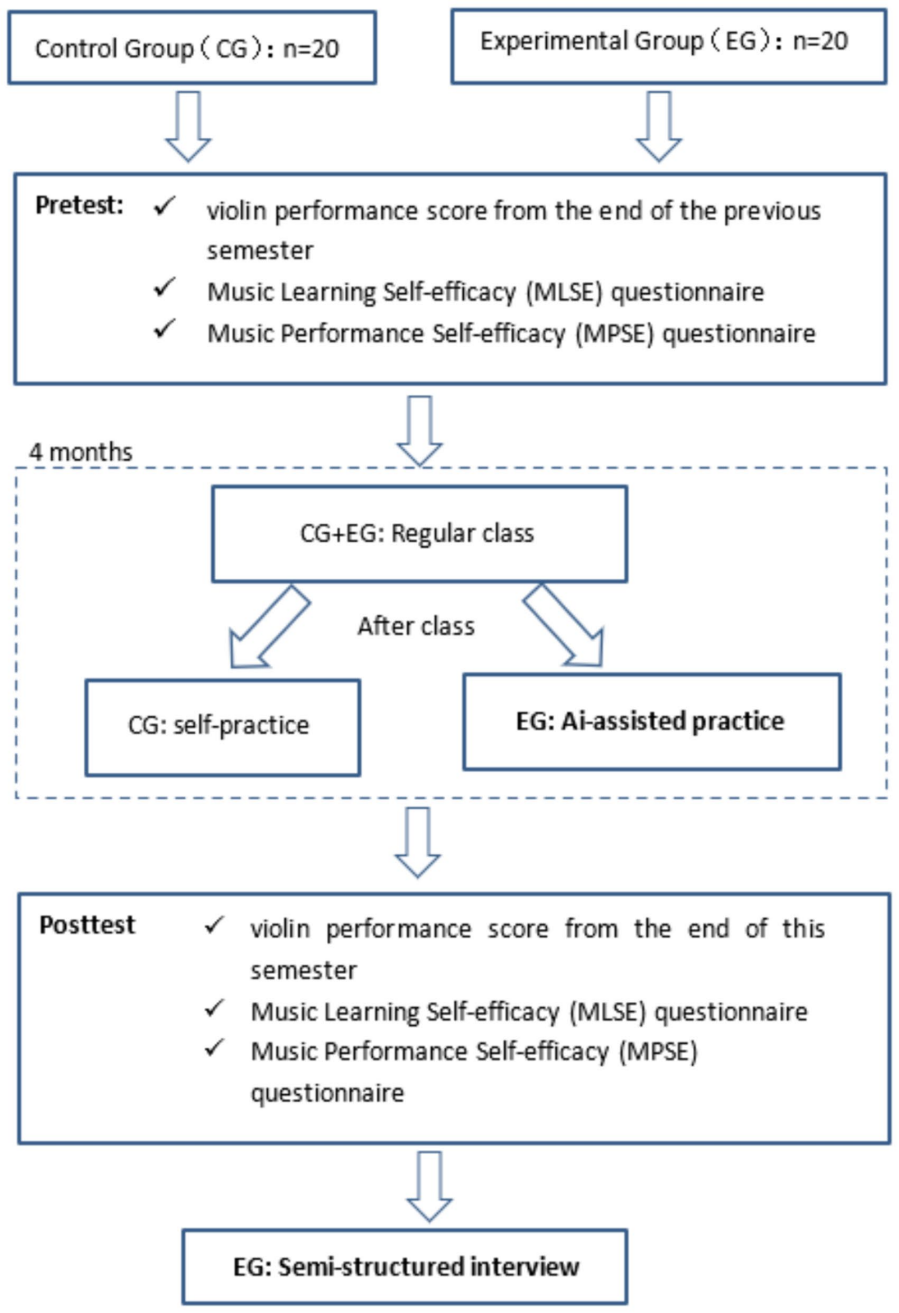
Research design and procedure.

The study design is illustrated in Figure 3. Both groups of students completed identical pre-tests via electronic questionnaires, which included filling out the MLSE and MPSE scales, as well as reporting their violin performance scores from the previous semester’s final exam (January 29, 2023).

Participants in the experimental group received a one-hour training session on the AI-assisted violin practice app prior to the experiment. They were assisted with software installation and granted VIP access to all AI features.

Starting from March 2023, for the following 4 months, all participants continued their regular coursework and individual practice routines (3–5 h per day). The only difference was in their post-class practice sessions: while control group students practiced on their own, experimental group students used Violy for assisted violin practice.

The experiment concluded at the end of the semester (early July 2023). We collected all 40 participants’ final exam violin performance scores and readministered the MLSE and MPSE questionnaires. Following the post-test, one-on-one semi-structured interviews were conducted with 10 students from the experimental group to collect qualitative data.

### 4.5 Data collection and analysis

#### 4.5.1 Quantitative data

Pre-test data were collected via Questionnaire Star, with all participants completing the MLSE and MPSE questionnaires on their smartphones and reporting violin performance scores from the previous semester (late January 2023). Post-test data were collected using the same platform, with participants reporting current semester scores (early July 2023) and completing the same questionnaires. Both phases achieved 100% response and validity rates. All performance scores were verified against official records as described in the methodology section.

Statistical analyses were conducted using SPSS version 26.0. Prior to analysis, reverse-scored items in the scale were recoded. Shapiro–Wilk tests revealed non-normal distributions for some variables, violating assumptions for traditional repeated-measures ANOVA. Therefore, separate linear mixed-effects models (LMMs) were fitted for each dependent variable. LMMs were selected because they are more robust to violations of sphericity and normality assumptions while accounting for individual variability through random effects (Hesselmann, 2018; Schielzeth et al., 2020). They also perform well with small sample sizes (Maas and Hox, 2005).

For each of the three dependent variables (MLSE, MPSE, and performance), the LMM model included group (control vs. experimental) and time (pre-test vs. post-test) as fixed effects, with participant ID as a random effect to account for within-subject correlation. As prior studies have demonstrated that these three dependent variables are typically moderately to highly correlated (Ou and Qin, 2025), a Bonferroni correction was applied to control for Type I error inflation due to multiple comparisons, with the significance level adjusted to α = 0.017 (0.05/3).

#### 4.5.2 Qualitative data

To explore participants’ subjective experiences, semi-structured interviews were conducted with 10 experimental group students after the experiment (see Appendix). Research indicates that 6–12 interviews typically achieve thematic saturation in homogeneous groups (Braun and Clarke, 2021), with most core themes emerging within the first 10 interviews (Namey et al., 2016).

Interviews were conducted via Tencent Meeting, lasting approximately 30 min each. Participants provided informed consent for recording. Recordings were transcribed using professional software, verified by two researchers, and confirmed by participants to ensure accuracy.

Interview transcripts were imported into NVivo as individual case files. Given the semi-structured nature of the interviews, we used SRL theory framework and elements to code the transcripts rather than following grounded theory’s three-stage approach.

The thematic analysis followed the six-phase approach outlined by (Braun and Clarke, 2014). The process began with familiarization with the data through repeated reading of the interview transcripts. Initial codes were then generated using a top-down approach, with parent nodes created based on the phases of SRL theory (Zimmerman, 2002). This was followed by a bottom-up analytical coding process to identify child nodes representing specific elements within each SRL phase, which were organized accordingly under their respective parent nodes. Subsequently, themes were reviewed, refined, and defined through careful examination of all coded data. Nodes with similar or overlapping meanings were merged, and the hierarchical structure was adjusted to ensure conceptual clarity and coherence. An example of the coding process is presented in Table 5. Coding was conducted by one researcher and subsequently reviewed and validated by the first author.

**Table 5:** Coding examples.

First-level node	Second-level node	Third-level node	Material source count	Interviewer answer
Outcome	Skill Improvement	Improve the pitch	5	“…… *And then in the past four months I think my pitch and rhythmic stability has improved very much.” (This statement indicates both improved pitch and rhythm, so it was coded as both “improved pitch” and “stabilized rhythm*.”*)*
		Stable the rhythm	4	“…… *It helps me to remember the whole framework of the music when I’m doing the music processing in post, and to play along with the beat without changing the overall framework, rather than changing the tempo haphazardly, this is the highlight where I think it’s helped me a lot.*”
		Improved sight-reading ability	1	“…… *And now that I’m playing other pieces, I feel significantly more relaxed, I can read music faster, and I’m able to memorize the music quickly when auditioning* …”

## 5 Results

### 5.1 Linear mixed-effects model for MLSE

For MLSE, the Linear Mixed-Effects analysis revealed no significant main effect of time, *F* (1, 38) = 0.773, *p* = 0.385, η^2^_p_ = 0.020. The main effect of group was significant before correction, *F* (1, 38) = 5.673, *p* = 0.022, η^2^_p_ = 0.130, but became non-significant after Bonferroni correction (*α* = 0.017) for multiple comparisons. Most importantly, a significant time × group interaction emerged, *F* (1, 38) = 18.149, *p* < 0.001, η^2^_p_ = 0.323, which remained highly significant after Bonferroni correction (*p* < 0.017). This interaction indicated that the two groups exhibited different patterns of change from pre-test to post-test, with a large effect size according to Cohen’s (1988) guidelines (Cohen, 1988).

To decompose the significant interaction, simple effects analyses were conducted. For the simple effect of group at each time point, no significant difference was found between groups at pre-test (experimental group: M = 5.20, SD = 1.28; control group: M = 5.45, SD = 0.92), *F* (1, 76) = 0.609, *p* = 0.437, d = 0.18, confirming baseline equivalence with a minimal effect size. This finding is consistent with our matching procedure described in the method section. At post-test, the experimental group (M = 5.87, SD = 0.81) significantly outperformed the control group (M = 4.43 SD = 0.98), *F* (1, 76) = 20.368, *p* < 0.001, d = 1.03, with a substantial mean difference of 1.44 points (95% CI [0.808, 2.083]) and a large effect size that remained highly significant after Bonferroni correction.

For the simple effect of time within each group, the control group showed a significant decrease from pre-test (M = 5.45, SD = 0.92) to post-test (M = 4.43, SD = 0.98), *F* (1, 76) = 10.197, *p* = 0.002, d = −0.74, representing a decline of 1.02 points with a medium-to-large effect size that remained significant after Bonferroni correction. The experimental group demonstrated a numerical increase from pre-test (M = 5.20, SD = 1.28) to post-test (M = 5.87, SD = 0.81), representing an improvement of 0.67 points, *F* (1, 76) = 4.412, *p* = 0.039, d = 0.49. While this moderate improvement did not achieve statistical significance after Bonferroni correction (α = 0.017), the overall pattern of results still supports the positive impact of AI-assisted practice app use on MLSE.

**Figure 4:**
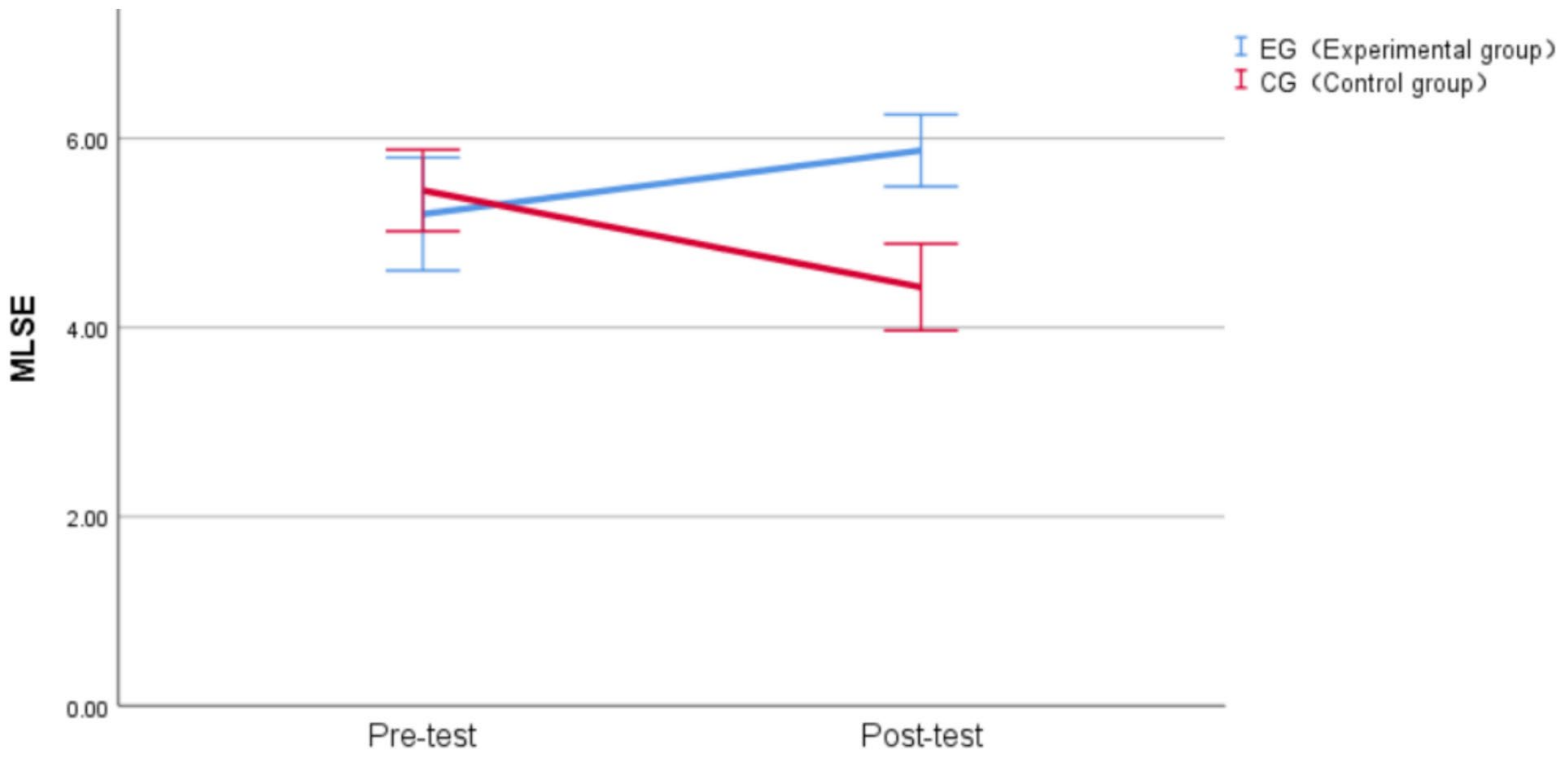
Group × Time interaction effects on MLSE. Vertical bars denote 95% confidence intervals.

Specifically, when considered in conjunction with the significant decline observed in the control group, the experimental group’s maintenance and numerical improvement suggests a protective or beneficial effect of AI intervention. The experimental group demonstrated a moderate improvement (d = 0.49), while the control group showed a significant decline. The interaction between Group and Time on MLSE is presented in Figure 4.

### 5.2 Linear mixed-effects model for MPSE

The Linear Mixed-Effects analysis revealed no significant main effect of time, *F* (1, 38) = 0.086, *p* = 0.771, η^2^_p_ = 0.002. The main effect of group was significant before correction, *F* (1, 38) = 10.812, *p* = 0.002, η^2^_p_ = 0.221, but became non-significant after Bonferroni correction (α = 0.017) for multiple comparisons. A significant time × group interaction emerged, *F* (1, 38) = 20.378, *p* < 0.001, η^2^_p_ = 0.349, which remained highly significant after Bonferroni correction. This interaction indicated that the two groups exhibited different patterns of change from pre-test to post-test for MPSE, with a large effect size.

To decompose the significant interaction, simple effects analyses were conducted. For the simple effect of group at each time point, no significant difference was found between groups at pre-test (experimental group: M = 4.89, SD = 1.50; control group: M = 4.72, SD = 1.11), *F* (1, 76) = 0.197, *p* = 0.658, d = 0.10, confirming baseline equivalence with a trivial effect size. This finding aligns with our matching procedure, ensuring comparable baseline MPSE levels across groups. However, at post-test, the experimental group (M = 5.79, SD = 0.79) significantly outperformed the control group (M = 3.92, SD = 1.08), *F* (1, 76) = 26.632, *p* < 0.001, d = 1.18, with a substantial mean difference of 1.87 points (95% CI [1.150, 2.595]) and a large effect size that remained highly significant after Bonferroni correction.

For the simple effect of time within each group, the control group showed a decrease from pre-test (M = 4.72, SD = 1.11) to post-test (M = 3.92, SD = 1.08) of 0.8 points, *F* (1, 76) = 4.856, *p* = 0.030, d = −0.51, though this decline was not statistically significant after Bonferroni correction. The experimental group showed a significant increase from pre-test (M = 4.88, SD = 1.50) to post-test (M = 5.79, SD = 0.79) of 0.911 points, *F* (1, 76) = 6.289, *p* = 0.014, d = 0.58, which remained significant after correction, representing a medium-to-large effect size.

**Figure 5:**
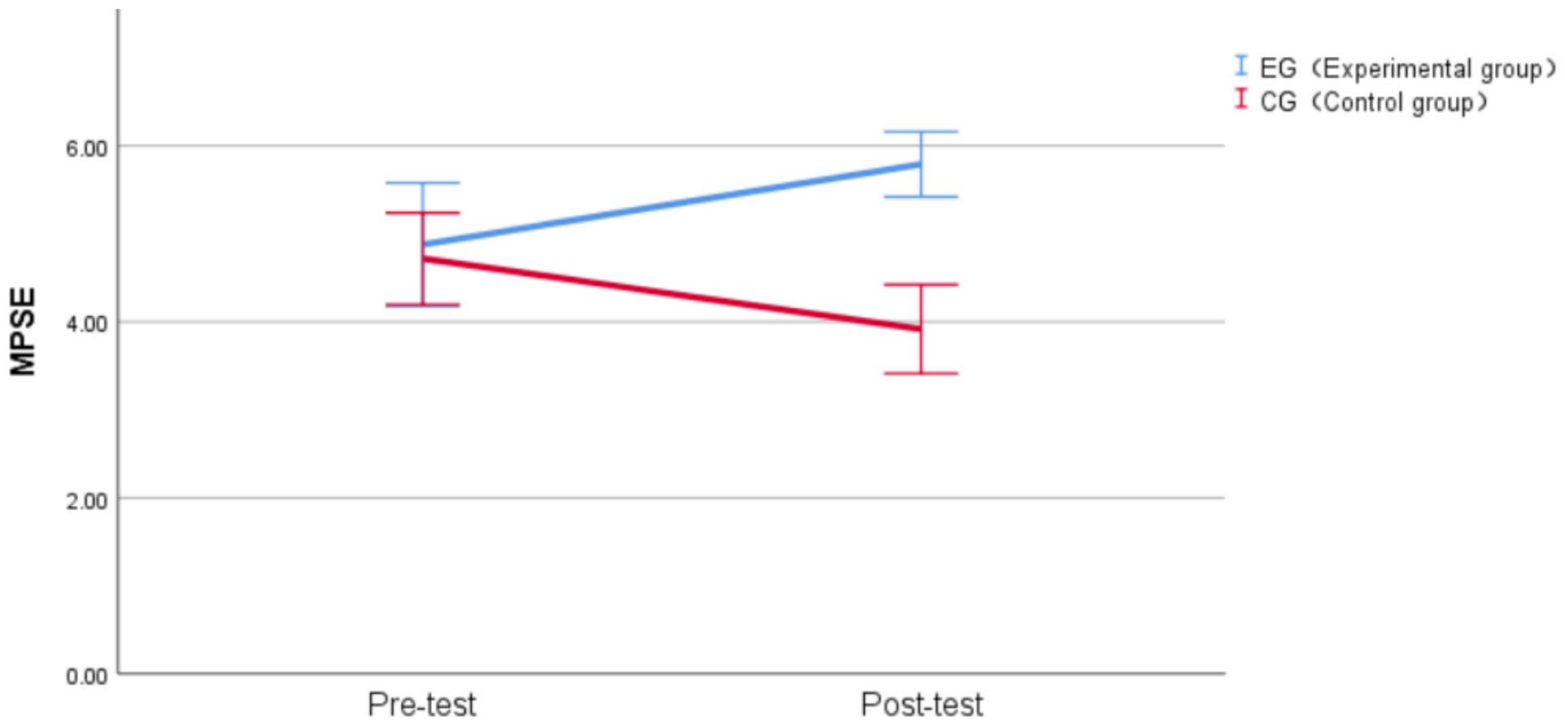
Group × Time interaction effects on MPSE. Vertical bars denote 95% confidence intervals.

Unlike the pattern observed for MLSE, the experimental group demonstrated a significant improvement in MPSE performance (d = 0.58), while the control group showed a non-significant decline. The interaction between group and time on MPSE is presented in Figure 5. The significant interaction effect (η^2^_p_ = 0.349) demonstrates that AI-assisted practice app had a particularly strong facilitative effect on MPSE, with the post-test group difference (d = 1.18) representing a substantial practical advantage for the AI-assisted learning condition.

### 5.3 Linear mixed-effects model for music performance

The analysis revealed no significant main effect of time, *F* (1, 38) = 0.659, *p* = 0.422, η^2^_p_ = 0.017. The main effect of group was significant, *F* (1, 38) = 9.782, *p* = 0.003, η^2^_p_ = 0.205, and remained significant after Bonferroni correction (*α* = 0.017), suggesting overall group differences across time points. A significant time × group interaction emerged, *F* (1, 38) = 7.618, *p* = 0.009, η^2^_p_ = 0.167, which remained significant after Bonferroni correction. This interaction indicated that the two groups exhibited different patterns of change from pre-test to post-test for performance, with a medium-to-large effect size.

Simple effects analyses revealed no significant difference between groups at pre-test (experimental group; control group: M = 85.25, SD = 2.02; M = 84.70, SD = 1.56), *F* (1, 72.198) = 0.546, *p* = 0.462, d = 0.18, confirming baseline equivalence. This result is consistent with our matching procedure. However, at post-test, the experimental group (M = 86.90, SD = 3.04) significantly outperformed the control group (M = 83.80, SD = 2.53), *F* (1, 72.198) = 17.350, *p* < 0.001, d = 1.01, with a substantial mean difference of 3.1 points (95% CI [1.616, 4.584]) and a large effect size that remained highly significant after Bonferroni correction.

For the simple effect of time within each group, the control group showed a decrease from pre-test (M = 84.70, SD = 1.56) to post-test (M = 83.80, SD = 2.53) of 0.9 points, *F* (1, 38) = 1.898, *p* = 0.176, d = −0.32, though this decline was not statistically significant after Bonferroni correction. The experimental group showed a significant increase from pre-test (M = 85.25, SD = 2.02) to post-test (M = 86.90, SD = 3.04) of 1.65 points, *F* (1, 38) = 6.379, *p* = 0.016, d = 0.58, which remained significant after correction, representing a medium-to-large effect size.

**Figure 6:**
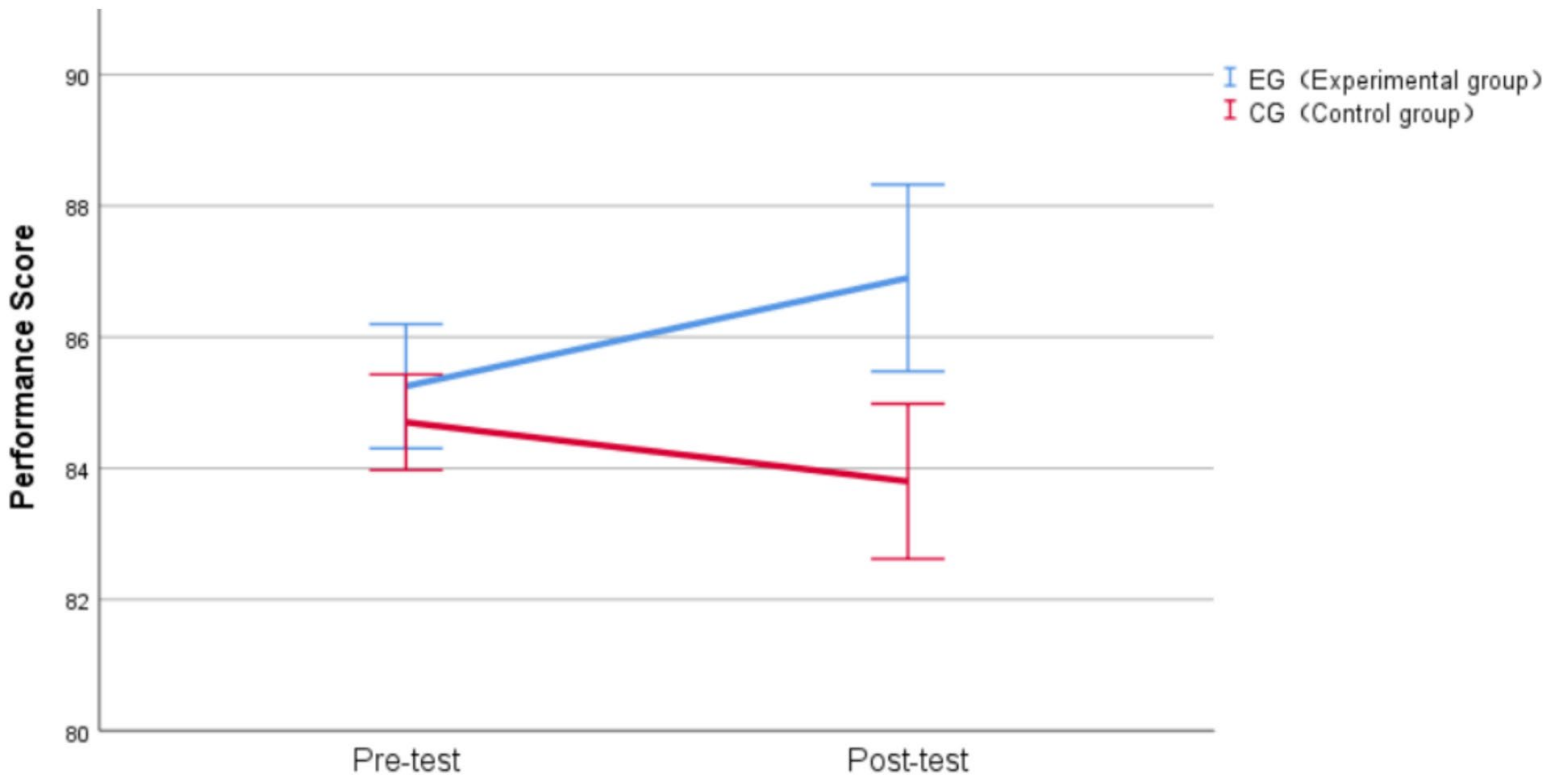
Group × Time interaction effects on performance. Vertical bars denote 95% confidence intervals.

The experimental group demonstrated a significant improvement in performance (d = 0.58), while the control group showed a non-significant decline. The interaction between group and time on performance is presented in Figure 6. The significant interaction effect (η^2^_p_ = 0.167) demonstrates that AI-assisted practice app had a facilitative effect on performance, with the post-test group difference (d = 1.01) representing a substantial practical advantage for the AI-assisted learning condition.

### 5.4 Student perspectives on AI-assisted practice

The interview results indicated that participants in experimental group practiced the violin for 3–5 h each day. Within this daily practice time, 1–2 h involved the use of AI-assisted apps. The features they found most valuable included: Audition Report (mentioned by 7 students), Daily Challenge (Check-in Function) (4 students), Accompaniment function (3 students), Note-by-Note (3 students), and Video Demonstrations (2 students). They felt the AI app most significantly helped their violin skills by improving intonation (5), stabilizing rhythm (4), and enhancing sight-reading ability (1). After about a month of using the app, they began to notice improvements in their playing, which in turn boosted their performance confidence (10).

When the students explained how they used the app’s features, the impact of various functions of the AI app on their SRL was observed.

#### 5.4.1 Forethought phase

The AI-assisted practice app supports students’ goal-setting and strategic planning during the forethought phase through multiple features. The Video Demonstration function provides clear models for students to observe and imitate, facilitating effective goal-setting by showcasing performance videos of other musicians or students. This allows students to learn from outstanding playing techniques and establish realistic practice objectives.

*“The demonstration function is also quite effective, as it displays the sheet music right below the video on the screen. For instance, when a teacher assigns a new piece, you can listen to the music and watch the demonstration and instructional videos directly on the app. This helps you gain a better understanding of the piece. In the initial stages of practice, this approach can greatly enhance efficiency” (Student 8#).*

The AI Assessment function further supports strategic planning by helping students identify specific areas requiring attention. Based on continuous error and score records from previous sessions, students can form objective self-assessments of their playing level, enabling them to set reasonable and targeted goals for future practice sessions.

*“For example, I set a goal to focus on the fast sections of pages three and four of a piece, then set it up on the tablet. I practice, continuously correcting the wrong notes. After an hour of practice, I’ve resolved these pages. To me, this means I’ve achieved my goal for today. In the afternoon, I’ll work on pages five and six, and by the end of the day, I’ll have played through many pages of Brahms with much more proficiency and fewer mistakes” (Student 10#).”*

*“……The biggest difference is that the app can guide me on the right direction for practice, giving me a general goal to aim for” (Student 4#).*

#### 5.4.2 Performance phase

During the performance phase, the app provides real-time monitoring and immediate feedback to support students’ active practice engagement. Note-by-note (pitch-tracking function) instantly identify issues with pitch, rhythm, and tempo, marking incorrect spots with red dots to help students precisely locate areas for improvement while they practice.

*“… It’s really detailed, down to every note or rhythm. In the past, when I practiced on my own, I might not have been able to hear the mistakes, or I would just move past them. But with this app, it can precisely pinpoint every note and even tell me if it’s accurate or if the rhythm is correct, and I can immediately know” (Student 10#).*

This objective evaluation system eliminates human bias and provides clear, immediate feedback during practice sessions.

*“We are also invited by our colleagues to help listen. But sometimes we are hesitant to point out if they are playing correctly due to concerns about politeness. However, with this AI app, it clearly highlights mistakes with a red indicator, or when they follow along with the audio, they can realize what the overall effect of the piece should sound like” (Student 6#).*

The Daily Challenge (check-in function) uses sound detection to monitor actual violin practice time rather than mere app usage, ensuring genuine accountability during the performance phase. This system rewards students with collectible stars based on practice duration, which unlock progressive rewards (moons and suns) and serve as immediate motivational tools that provide instant feedback, prevent distractions, and sustain engagement throughout the practice process.

*“……Isn’t there a feature that records the duration of practice? I find this really helpful because it only records when there’s sound, which reduces the chances of me playing with my phone while practicing……” (Student 3#).*

*“……if I practice, it rewards me with a small star every 10 min. To earn these reward stars, I’ll keep practicing continuously. This helps prevent me from getting distracted or checking my phone or doing other things. As a result, it directly improves my focus during the entire practice process, and naturally, my overall practice efficiency increases” (Student 2#).*

*“It has a practice check-in feature where you earn a star if you practice for more than 10 min. Collecting a certain number of stars unlocks a moon and even the sun. This makes the process very specific and fun” (Student 9#).*

#### 5.4.3 Self-reflection phase

The app’s features strongly support the self-reflection phase by providing comprehensive performance records and fostering positive emotions through visible progress tracking. The Audition report function uses AI-based assessment and scoring to mark errors that reflect students’ daily performance, enabling effective self-monitoring, while systematically recorded session scores allow students to track their improvement over time.

*“……I use the scores to assess my level, which helps me correct mistakes during each practice session and prevent them from happening again” (Student 1#).*

*“……For example. Today I scored 75 points, and then tomorrow I focus on solving the specific issues and correct them. As a result, I get 88 points. I can see my progress every day. When I go to my teacher next week and realize that the problem has actually been solved, it makes me even happier” (Student 10#).*

The visible progress tracking creates positive emotional experiences and enhances both self-satisfaction and self-efficacy as students witness their daily improvements and receive recognition from teachers.

*“……After using the AI app, you can feel that you are improving every day. At this point, you start to want to showcase the changes you have made. For example, the teacher might say your rhythm has improved or your pitch is better. That makes you really happy, and it gives you even more motivation to practice when you go back home” (Student 9#).*

Furthermore, the Audition report function generates performance data that subsequently feeds into the forethought phase as previous performance records, enabling informed practice planning and strategic goal adjustment, thereby establishing a continuous self-regulated learning cycle where reflective insights drive future planning and sustained improvement.

## 6 Discussion

This study investigated the effects of an AI-assisted practice app on violin students’ learning. The findings suggest that the AI-assisted practice app significantly improves students’ musical performance and self-efficacy through systematic support of the self-regulated learning process.

Specifically, the study found significant improvements in MLSE (RQ1) and MPSE (RQ2), along with enhanced performance (RQ3). Qualitative analysis revealed that these improvements occurred through the app’s support of key self-regulatory processes, including goal setting, self-monitoring, and feedback utilization (RQ4). The following sections examine these findings in detail.

### 6.1 Impact on MLSE, MPSE and performance

This study revealed distinct patterns of AI intervention effects on the two dimensions of musical self-efficacy. For MLSE, the control group showed a significant decline (d = −0.74), while the experimental group remained stable with numerical improvement (d = 0.49). Research suggests that self-efficacy declines as students perceive tasks as more challenging (Lodewyk and Winne, 2005). This pattern is particularly evident among violin students who encounter progressively difficult repertoire and technical demands in new semester. McPherson and McCormick's (2006) longitudinal study confirmed this trend in musical learning, showing that students’ self-efficacy consistently decreased as difficulty increased—a finding supported by this study’s control group results.

However, AI intervention effectively countered this natural decline, aligning with recent findings on technology-supported learning. Panadero and Lipnevich (2022) demonstrated that timely personalized feedback maintains learners’ self-efficacy during challenging tasks. Similarly, our study’s AI-assisted app provided real-time error detection and step-by-step guidance, creating continuous successful experiences that sustained students’ confidence in their musical abilities.

More remarkably, the experimental group showed improvement in MPSE (d = 0.58), with the between-group difference reaching a large effect size (d = 1.18). This finding supports Schunk and Pajares (2009) perspective on the impact of specific, immediate feedback on task-specific self-efficacy. Unlike the vague subjective evaluations in traditional music instruction, the AI-assisted app provided objective, quantified performance feedback for each practice session. This “performance-oriented” feedback mechanism proved particularly effective in building performance confidence.

The significant improvement in actual performance among the experimental group (d = 0.58) validates the applicability of AI technology in enhancing musical instrumental learning environments. The maintenance of MLSE provided students with a motivational foundation for continued learning, while the significant improvement in MPSE directly translated into better performance outcomes. Clark’s (2012) research indicates that learners with high self-efficacy are more inclined to adopt cognitively complex and structured practice strategies, thereby achieving superior learning results. This study found a similar mechanism: AI intervention not only directly provided technical guidance but may also have motivated students to adopt more systematic and effective practice methods through the dual pathways of maintaining MLSE and enhancing MPSE. This enhancement of self-efficacy formed a virtuous cycle with the immediate feedback provided by the AI-assisted app, both reinforcing students’ technical skill development and cultivating the learning confidence necessary for continuous improvement, ultimately manifesting as measurable significant improvements in objective performance assessments.

Our quantitative analysis demonstrates that AI-assisted practice showed significant positive effects on student self-efficacy and performance outcomes in the Chinese conservatory context. However, caution is warranted when interpreting broader applicability. Our participants represent a specific cultural context where students traditionally rely heavily on teacher feedback with limited access to immediate, objective assessment during individual practice. AI applications may fill this gap by providing instant feedback, potentially contributing to the observed effects. Whether findings would generalize to educational cultures emphasizing student self-exploration and peer evaluation remains unclear, requiring future cross-cultural comparative studies.

### 6.2 Support mechanisms for SRL

**Figure 7:**
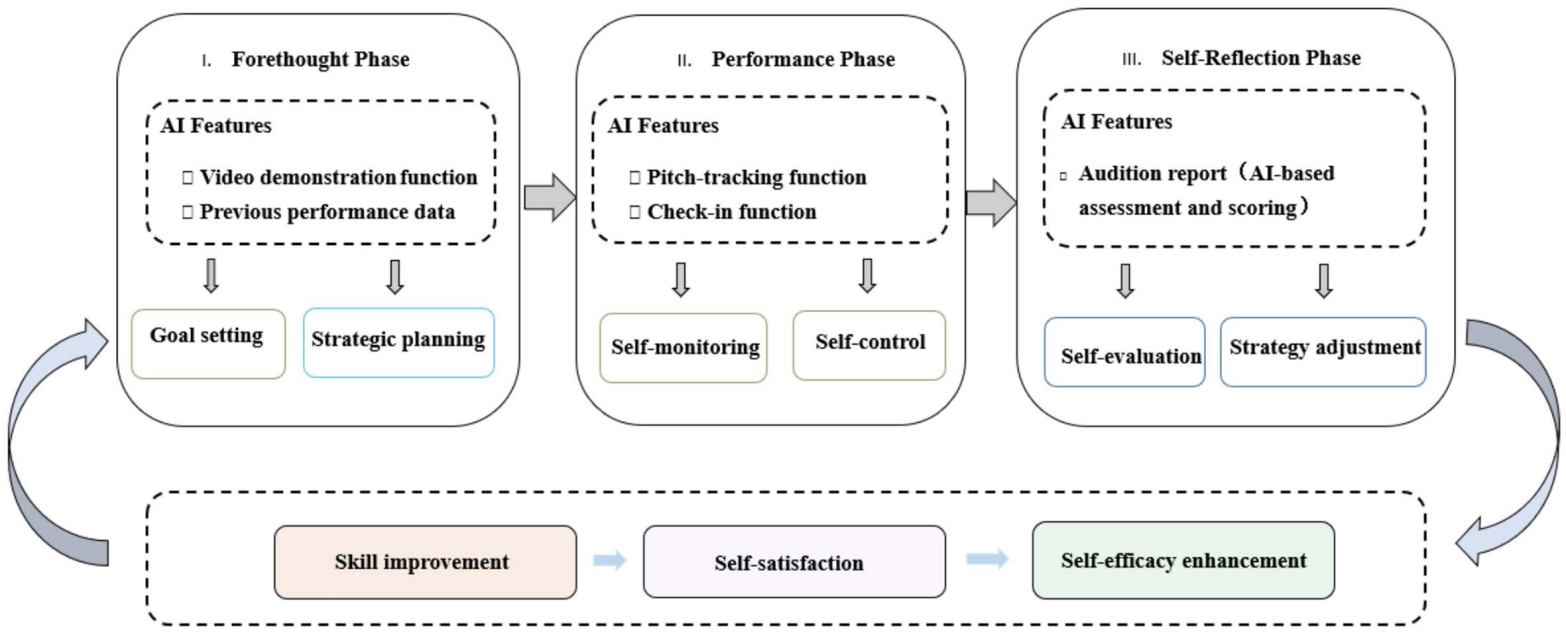
Mechanistic diagram of AI applications in enhancing on SRL.

Based on Zimmerman’s (2002) three-phase model of self-regulated learning, different AI-assisted functions provided comprehensive support for the learning process (see Figure 7).

In the forethought phase, the video demonstration function supported the goal-setting process by providing students with clear technical models. Students used these demonstrations as reference benchmarks to clarify specific technical and musical objectives. Meanwhile, the objective nature of historical performance data further enhanced the effectiveness of strategic planning. Students reported being able to utilize historical error patterns and scoring trends to identify specific technical areas requiring focused attention, thereby developing more targeted practice plans. This data-driven goal-setting approach represented a significant improvement over traditional practice modes that relied on subjective impressions or limited teacher feedback.

In the performance phase, real-time error detection and check-in functions provided strong support for self-monitoring and self-control. Student reports indicated that visualized error markers helped them maintain focus on key technical elements during performance and enabled real-time adjustment of practice strategies. This immediate feedback loop is crucial for effective self-regulated learning (Butler and Winne, 1995). The objectivity of AI feedback effectively addressed common cognitive bias issues among learners, including self-deception or excessive self-criticism that hinder learning progress (Dunning et al., 2004). Student reports indicated that objective data helped them develop a more balanced and realistic perception of their abilities, thereby enabling more effective selection of practice strategies and adjustment of learning goals.

The system precisely tracked actual practice time through audio detection technology rather than simple app usage duration, providing authentic and reliable practice supervision. The Daily Challenge function effectively addressed issues of practice motivation and persistence, which are core components of volitional control within the SRL framework (Zimmerman, 2002). It achieved this primarily through its gamification elements; the star reward system, for example, served as an external motivational factor that encouraged sustained participation and, over time, helped cultivate intrinsic motivation for self-improvement.

For many students, the self-reflection phase showed clear enhancement through AI scoring and assessment functions, providing strong support for systematic self-evaluation and strategic adjustment. Traditional violin practice, due to the transient nature of musical performance and the absence of objective feedback mechanisms, often struggles to establish effective reflection cycles. The AI scoring system and progress visualization functions provided students with concrete, quantifiable self-assessment data, enabling the reflection process to transcend the limitations of subjective impressions.

AI-assisted app’s support across all three phases of Zimmerman’s (2002) self-regulated learning model created a comprehensive learning environment that improved students’ performance skills, generated self-satisfaction from their progress, and ultimately enhanced their self-efficacy.

Previous research across second language learning, writing, and mathematics has demonstrated AI’s effectiveness in enhancing SRL processes (Wei, 2023; Chang and Sun, 2024), indicating broad cross-disciplinary applicability. Our study extends these findings to music education, providing empirical evidence of AI’s role in supporting SRL within this domain. The AI-supported SRL model addresses traditional music teaching limitations, such as delayed and subjective feedback (Wang et al., 2025), enabling students to develop stronger self-efficacy through enhanced practice experiences. This enriches SRL theory application in AI-enhanced music learning and demonstrates AI’s potential to contribute to the study of self-regulation in the instrumental domain by providing rich, objective data on how learners manage their cognitive and psychomotor processes during practice.

### 6.3 Limitations

While this study provides preliminary evidence for the effectiveness of AI-assisted music learning, several limitations need to be addressed in future research. First, the study duration was one semester (4 months), limiting our ability to assess long-term retention of performance improvements or sustained impacts on self-efficacy and performance outcomes. The novelty effect of AI technology may have influenced short-term results, and it remains unclear whether observed benefits would persist over extended periods. Second, this research focused on a specific cohort—violin majors from a conservatory in South China—which limits the generalizability of the findings across different instrumental, educational, and cultural contexts. Third, the study recruited 40 participants, indicating a relatively small sample size. Additionally, among the recruited students, there was a gender imbalance, with males comprising 35% (14 participants) and females 65% (26 participants), which may introduce bias into the research findings.

Future research should conduct longitudinal follow-up studies tracking participants over extended periods (1–2 years) to examine the durability of self-efficacy and performance improvements. Additionally, studies should expand the participant pool with larger, more gender-balanced samples across different musical instruments and diverse educational and cultural contexts to investigate potential differential effects of AI-assisted interventions. Research is also needed to investigate optimal integration models between AI-assisted learning and traditional face-to-face instruction.

## 7 Conclusion

The research findings demonstrate that AI technology exhibits significant educational value in instrumental music learning. Regarding Music Learning Self-Efficacy (MLSE), AI intervention effectively prevented the natural decline that typically occurs in traditional learning environments as task difficulty increases. While the experimental group maintained stable learning confidence, the control group experienced significant deterioration. More notably, in the Music Performance Self-Efficacy (MPSE) dimension, the experimental group achieved significant improvement with a large between-group effect size, indicating that AI-assisted apps possess distinct advantages in enhancing learners’ performance confidence. The study reveals the differentiated impact mechanisms of AI intervention on both dimensions of musical self-efficacy, enriching our understanding of Bandura’s self-efficacy theory within technology-enhanced learning environments.

Furthermore, the experimental group achieved significant improvement in violin performance, while the control group showed a declining trend, creating a clear divergence in developmental patterns between the two groups. This confirms the substantive facilitative role of AI technology in musical skill development.

Thematic analysis of the interviews elucidated how AI technology supports three critical phases of SRL through different functional characteristics. During the forethought phase, it provides goal-setting and strategic planning support. In the performance phase, it enables real-time monitoring and immediate feedback through automated scoring systems and instant error detection algorithms. During the self-reflection phase, it facilitates objective evaluation and strategy adjustment by providing quantifiable performance data and systematic progress tracking records. This finding provides new insights into understanding how technology promotes autonomous learning of complex skills within Zimmerman’s self-regulated learning framework.

For music educators, this research suggests a need to reconsider the role positioning of AI tools in music learning. AI-assisted apps should not merely be viewed as technical training tools, but rather understood as comprehensive educational resources capable of influencing learning motivation, self-perception, and learning strategies.

## Data Availability

The raw data supporting the conclusions of this article will be made available by the authors, without undue reservation.

## Appendix

Interview outline.

**Table:**

Personal information	Gender, age, practice time per week
Practice time	(1) What time do you usually use Violy for practice?
	(2) How long do you use Violy each time?
	(3) Which functions of Violy do you use most frequently?
Ability	(1) Do you think using Violy has helped you improve your violin skills? I’d love to hear some examples!
	(2) Which functions of Violy do you find most helpful in improving your musical skills? Could you please provide me more information?
	(3) Which functions of Violy have helped you improve your practice efficiency?
Confidence	(1) Do you feel more confident in your practice while using Violy? I’d love to hear any examples you can share!
	(2) Do you feel more confident in your learning and practice with Violy? I am really interested to know if there was a moment when you noticed the biggest change!
	(3) Do you think there is anything about Violy’s reward system that could be improved to help boost your confidence even more? It would be great to hear any thoughts or ideas you have!
	(4) How do you think using Violy for practice has helped with your mental and physical state during performances on stage?
Comparison	(1) Do you feel any difference between practicing with Violy and practicing on your own? I’d love to hear your thoughts on that!
	(2) What positive effects do you think this difference has brought you?

## References

[ref1] Ahola S. Malmberg J. Järvenoja H. (2023). Investigating the relation of higher education students’ situational self-efficacy beliefs to participation in group level regulation of learning during a collaborative task. Cogent Educ. 10:2164241. doi: 10.1080/2331186X.2022.2164241

[ref2] Antonini Philippe R. Kosirnik C. Vuichoud N. Clark T. Williamon A. McPherson G. E. (2020). Conservatory musicians’ temporal organization and self-regulation processes in preparing for a music exam. Front. Psychol. 11:89. doi: 10.3389/fpsyg.2020.00089, PMID: 32116910 PMC7008847

[ref3] Arum T. A. S. Jacob D. W. (2024). Exploring innovative music teaching management systems: a systematic literature review. Jurnal Syntax Admiration 5, 2589–2600. doi: 10.46799/jsa.v5i7.1302

[ref4] Banar B. (2025). Composing contemporary classical music using generative deep learning. Queen Mary University of London. Available online at: https://qmro.qmul.ac.uk/xmlui/handle/123456789/106333 (Accessed July 25, 2025).

[ref5] Bandura A. (1977). Self-efficacy: toward a unifying theory of behavioral change. Psychol. Rev. 84, 191–215. doi: 10.1037/0033-295X.84.2.191, PMID: 847061

[ref5000] Bandura A. (1986). Social foundations of thought and action: a social cognitive theory. Englewood Cliffs. NJ: Prentice-Hall, Inc., PMID: 847061

[ref6] Braun V. Clarke V. (2014). What can “thematic analysis” offer health and wellbeing researchers? Int. J. Qual. Stud. Health Well-being 9:26152. doi: 10.3402/qhw.v9.26152, PMID: 25326092 PMC4201665

[ref7] Braun V. Clarke V. (2021). To saturate or not to saturate? Questioning data saturation as a useful concept for thematic analysis and sample-size rationales. Qual. Res. Sport, Exerc. Health 13, 201–216. doi: 10.1080/2159676X.2019.1704846

[ref8] Butler D. L. Winne P. H. (1995). Feedback and self-regulated learning: a theoretical synthesis. Rev. Educ. Res. 65, 245–281. doi: 10.3102/00346543065003245

[ref9] Candusso S. (2024). Exploring the impact of generative AI on the music composition market: a study on public perception, behavior, and industry implications. Politecnico di Torino. Available online at: https://webthesis.biblio.polito.it/34172/ (Accessed July 25, 2025).

[ref10] Chang C.-Y. Panjaburee P. Lin H.-C. Lai C.-L. Hwang G.-H. (2022). Effects of online strategies on students’ learning performance, self-efficacy, self-regulation and critical thinking in university online courses. Educ. Technol. Res. Dev. 70, 185–204. doi: 10.1007/s11423-021-10071-y

[ref11] Chang W.-L. Sun J. C.-Y. (2024). Evaluating AI’S impact on self-regulated language learning: a systematic review. System 126:103484. doi: 10.1016/j.system.2024.103484

[ref12] Chen L. (2024). Delving into the role of self-efficacy in predicting motivation and engagement among music learners. Learn. Motiv. 86:101961. doi: 10.1016/j.lmot.2024.101961

[ref13] Chen L. Chen P. Lin Z. (2020). Artificial intelligence in education: a review. IEEE Access 8, 75264–75278. doi: 10.1109/ACCESS.2020.2988510

[ref14] Cheng Z. Southcott J. (2023). Practice and learning the piano: motivation and self-regulation. Int. J. Music. Educ. 41, 345–357. doi: 10.1177/02557614221125173

[ref15] Clark I. (2012). Formative assessment: assessment is for self-regulated learning. Educ. Psychol. Rev. 24, 205–249. doi: 10.1007/s10648-011-9191-6

[ref16] Coetzer J. S. (2024). The application of a seamless learning approach in the year 9 of the LGR-22 music curriculum using the SLED framework: a case study. University of the Free State. Available online at: http://hdl.handle.net/11660/13048 (Accessed July 24, 2025).

[ref17] Cohen J. (1988). Set correlation and contingency tables. Appl. Psychol. Meas. 12, 425–434. doi: 10.1177/014662168801200410

[ref18] Coluccio G. Muñoz-Herrera S. Adriasola E. Escobar E. (2024). Leadership development in women STEM students: the interplay of task behaviors, self-efficacy, and university training. Behav. Sci. 14:1087. doi: 10.3390/bs14111087, PMID: 39594387 PMC11591077

[ref19] Dai D. D. (2021). Artificial intelligence technology assisted music teaching design. Sci. Program. 2021, 1–10. doi: 10.1155/2021/9141339, PMID: 40995476

[ref20] Daubney A. Fautley M. (2020). Editorial research: music education in a time of pandemic. Br. J. Music Educ. 37, 107–114. doi: 10.1017/S0265051720000133

[ref21] Dong S. Gedvilienė G. (2025). Using self-efficacy and reflection to improve piano learning performance. Educ. Sci. 15:50. doi: 10.3390/educsci15010050

[ref22] Dos Santos Silva C. Marinho H. (2025). Self-regulated learning processes of advanced musicians: a PRISMA review. Music. Sci. 29, 89–108. doi: 10.1177/10298649241275614 PMC10864437 38356775

[ref23] Dunning D. Heath C. Suls J. M. (2004). Flawed self-assessment: implications for health, education, and the workplace. Psychol. Sci. Public Interest 5, 69–106. doi: 10.1111/j.1529-1006.2004.00018.x, PMID: 26158995

[ref24] Evin M. (2024). “A review on AI-enabled techniques for evaluating musician’s performance.” in AIP Conference Proceedings 3149, 140018.

[ref25] Faul F. Erdfelder E. Lang A.-G. Buchner A. (2007). G*power 3: a flexible statistical power analysis program for the social, behavioral, and biomedical sciences. Behav. Res. Methods 39, 175–191. doi: 10.3758/BF03193146, PMID: 17695343

[ref26] Gale J. Alemdar M. Cappelli C. Morris D. (2021). A mixed methods study of self-efficacy, the sources of self-efficacy, and teaching experience. Front. Educ. 6:750599. doi: 10.3389/feduc.2021.750599

[ref27] Gaunt H. Duffy C. Coric A. González Delgado I. R. Messas L. Pryimenko O.. (2021). Musicians as “makers in society”: a conceptual foundation for contemporary professional higher music education. Front. Psychol. 12:713648. doi: 10.3389/fpsyg.2021.713648, PMID: 34413817 PMC8368725

[ref28] Hasnine M. N. Nguyen H. T. Ueda H. (2024). Developing Taskcave system for task-based language teaching: teachers monitoring how students learn outside classroom. Procedia Comput. Sci. 246, 113–120. doi: 10.1016/j.procs.2024.09.233

[ref29] Hatfield J. L. Halvari H. Lemyre P.-N. (2017). Instrumental practice in the contemporary music academy: a three-phase cycle of self-regulated learning in music students. Music. Sci. 21, 316–337. doi: 10.1177/1029864916658342

[ref30] Hesselmann G. (2018). Applying linear mixed effects models (LMMs) in within-participant designs with subjective trial-based assessments of awareness—a caveat. Front. Psychol. 9:788. doi: 10.3389/fpsyg.2018.00788, PMID: 29887820 PMC5982570

[ref31] Jamshidi F. Marghitu D. Chapman R. (2021). “Developing an online music teaching and practicing platform via machine learning: a review paper” in Universal access in human-computer interaction. Access to media, learning and assistive environments. eds. Antona M. Stephanidis C. (Cham: Springer International Publishing), 95–108.

[ref32] Jiang J. (2024). Impact of music learning on students’ psychological development with mediating role of self-efficacy and self-esteem. PLoS One 19:e0309601. doi: 10.1371/journal.pone.0309601, PMID: 39226287 PMC11371213

[ref33] Kandemir Ö. Yokuş T. (2023). The effect of learning strategies on piano performance self-efficacy levels and performance success. Cukurova Univ. Faculty Educ. J. 52, 446–470. doi: 10.14812/cuefd.1265516

[ref34] Lin F. Yunus M. M. (2024). A systematic review of metaverse-based learning in music education. Int. J. Adv. Res. Fut. Ready Learn. Educ. 37, 41–58. doi: 10.37934/frle.37.1.4158

[ref35] Lodewyk K. R. Winne P. H. (2005). Relations among the structure of learning tasks, achievement, and changes in self-efficacy in secondary students. J. Educ. Psychol. 97, 3–12. doi: 10.1037/0022-0663.97.1.3

[ref36] López-Calatayud F. Tejada J. (2023). Self-efficacy in instrumental practice with intonation assessment software: a multiple case study with novice violin and viola students. J. Music Technol. Educ. 16, 57–76. doi: 10.1386/jmte_00060_1

[ref37] López-Calatayud F. Tejada J. (2024). Self-regulation strategies and behaviors in the initial learning of the viola and violin with the support of software for real-time instrumental intonation assessment. Res. Stud. Music Educ. 46, 48–65. doi: 10.1177/1321103X221128733

[ref38] López-Íñiguez G. McPherson G. E. (2020). Applying self-regulated learning and self-determination theory to optimize the performance of a concert cellist. Front. Psychol. 11:385. doi: 10.3389/fpsyg.2020.00385, PMID: 32210895 PMC7067924

[ref39] López-Íñiguez G. McPherson G. E. (2024). Using a music microanalysis protocol to enhance instrumental practice. Front. Psychol. 15:1368074. doi: 10.3389/fpsyg.2024.1368074, PMID: 38629042 PMC11020086

[ref40] Lu L. (2025). Ai-powered intelligent music education systems for real-time feedback and performance assessment. Int. J. Inf. Commun. Technol. 26, 33–47. doi: 10.1504/IJICT.2025.146690

[ref41] Maas C. J. M. Hox J. J. (2005). Sufficient sample sizes for multilevel modeling. Methodology 1, 86–92. doi: 10.1027/1614-2241.1.3.86

[ref42] MacAfee E. (2021). Music performance anxiety, self-efficacy, and the effects of self-modeling on young musicians. Université d’Ottawa / University of Ottawa. Available online at: http://hdl.handle.net/10393/41875 (Accessed July 24, 2025).

[ref43] MacRitchie J. Garrido S. (2019). Ageing and the orchestra: self-efficacy and engagement in community music-making. Psychol. Music 47, 902–916. doi: 10.1177/0305735619854531

[ref44] McPherson G. E. McCormick J. (2006). Self-efficacy and music performance. Psychol. Music 34, 322–336. doi: 10.1177/0305735606064841

[ref45] McPherson G. E. Miksza P. Evans P. (2017). “Self-regulated learning in music practice and performance” in Handbook of self-regulation of learning and performance. eds. D. H. Schunk and J. A. Greene (New York, NY: Routledge/Taylor & Francis Group), 181–193. doi: 10.4324/9781315697048-12

[ref46] Miksza P. (2015). The effect of self-regulation instruction on the performance achievement, musical self-efficacy, and practicing of advanced wind players. Psychol. Music 43, 219–243. doi: 10.1177/0305735613500832

[ref47] Namey E. Guest G. McKenna K. Chen M. (2016). Evaluating bang for the buck: a cost-effectiveness comparison between individual interviews and focus groups based on thematic saturation levels. Am. J. Eval. 37, 425–440. doi: 10.1177/1098214016630406

[ref48] Nelson P. N. (2023). Formal training in musical instrument and effective performance on stage. J. Music. Perform. Arts. 5, 1–10.

[ref49] Nenadic E. (2023). Where is the learning between young people, teachers, and professional musicians? A study of learning cultures within three music education partnership projects in England. Birmingham City University. Available online at: https://www.open-access.bcu.ac.uk/14711/ (Accessed July 24, 2025).

[ref50] Nguyen H. Nguyen A. (2024). Reflective practices and self-regulated learning in designing with generative artificial intelligence: an ordered network analysis. J. Sci. Educ. Technol. (In Press). doi: 10.1007/s10956-024-10175-z

[ref51] Oliveira A. Ribeiro F. S. Ribeiro L. M. McPherson G. Oliveira-Silva P. (2021). Disentangling motivation within instrumental music learning: a systematic review. Music. Educ. Res. 23, 105–122. doi: 10.1080/14613808.2020.1866517

[ref52] Ou J. Qin C. (2025). Exploring musical self-efficacy and performance anxiety in young violin learners: insights from mainland China. Front. Psychol. 16:1575591. doi: 10.3389/fpsyg.2025.1575591, PMID: 40636046 PMC12239020

[ref53] Panadero E. Lipnevich A. A. (2022). A review of feedback models and typologies: towards an integrative model of feedback elements. Educ. Res. Rev. 35:100416. doi: 10.1016/j.edurev.2021.100416

[ref54] Regier B. J. (2022). High school jazz band directors’ efficacious sources, self-efficacy for teaching strategies, and pedagogical behaviors. J. Res. Music. Educ. 70, 92–108. doi: 10.1177/00224294211024530

[ref55] Ritchie L. Williamon A. (2011). Measuring distinct types of musical self-efficacy. Psychol. Music 39, 328–344. doi: 10.1177/0305735610374895

[ref56] Roberts S. (2024). Factors shaping secondary music teachers’ student self-assessment practices in music performance pedagogy: an ecological framework. CQUniversity.

[ref57] Sajja R. Sermet Y. Cikmaz M. Cwiertny D. Demir I. (2024). Artificial intelligence-enabled intelligent assistant for personalized and adaptive learning in higher education. Information 15:596. doi: 10.3390/info15100596

[ref58] Schielzeth H. Dingemanse N. J. Nakagawa S. Westneat D. F. Allegue H. Teplitsky C.. (2020). Robustness of linear mixed-effects models to violations of distributional assumptions. Methods Ecol. Evol. 11, 1141–1152. doi: 10.1111/2041-210X.13434

[ref59] Schunk D. H. Mullen C. A. (2012). “Self-efficacy as an engaged learner” in Handbook of research on student engagement. eds. Christenson S. L. Reschly A. L. Wylie C. (Boston, MA: Springer US), 219–235.

[ref60] Schunk D. H. Pajares F. (2009). “Self-efficacy theory” in Handbook of motivation at school eds. K. R. Wentzel and A. Wigfield (New York: Routledge), 35–53.

[ref61] Spahn C. Tenbaum P. Immerz A. Hohagen J. Nusseck M. (2023). Dispositional and performance-specific music performance anxiety in young amateur musicians. Front. Psychol. 14:1208311. doi: 10.3389/fpsyg.2023.1208311, PMID: 37583605 PMC10425269

[ref62] Taylor K. A. C. (2021). An investigation of musicians’ goals as motivators and regulators. Royal College of Music. Available online at: https://researchonline.rcm.ac.uk/id/eprint/1811/ (Accessed July 24, 2025).

[ref63] Tsao J. Nogues C. (2024). Beyond the author: artificial intelligence, creative writing and intellectual emancipation. Poetics 102:101865. doi: 10.1016/j.poetic.2024.101865

[ref64] Wang X. (2022). Design of vocal music teaching system platform for music majors based on artificial intelligence. Wirel. Commun. Mob. Comput. 2022:5503834. doi: 10.1155/2022/5503834

[ref65] Wang X. Li P. (2024). Assessment of the relationship between music students’ self-efficacy, academic performance and their artificial intelligence readiness. Eur. J. Educ. 59:e12761. doi: 10.1111/ejed.12761

[ref66] Wang W.-S. Lin C.-J. Lee H.-Y. Huang Y.-M. Wu T.-T. (2025). Enhancing self-regulated learning and higher-order thinking skills in virtual reality: the impact of ChatGPT-integrated feedback aids. Educ. Inf. Technol. 30, 19419–19445. doi: 10.1007/s10639-025-13557-x

[ref67] Wei L. (2023). Artificial intelligence in language instruction: impact on english learning achievement, L2 motivation, and self-regulated learning. Front. Psychol. 14:1261955. doi: 10.3389/fpsyg.2023.1261955, PMID: 38023040 PMC10658009

[ref68] Zarza-Alzugaray F. J. Casanova O. McPherson G. E. Orejudo S. (2020). Music self-efficacy for performance: an explanatory model based on social support. Front. Psychol. 11:1249. doi: 10.3389/fpsyg.2020.01249, PMID: 32670146 PMC7330084

[ref69] Zeidner M. Stoeger H. (2019). Self-regulated learning (SRL): a guide for the perplexed. High Abil. Stud. 30, 9–51. doi: 10.1080/13598139.2019.1589369

[ref70] Zelenak M. S. (2024). Self-efficacy and music performance: a meta-analysis. Psychol. Music 52, 649–667. doi: 10.1177/03057356231222432

[ref71] Zimmerman B. J. (2002). Becoming a self-regulated learner: an overview. Theory Into Pract. 41, 64–70. doi: 10.1207/s15430421tip4102_2

